# Veterinary Association of the District of Columbia

**Published:** 1897-05

**Authors:** J. P. Turner

**Affiliations:** Secretary


Secretary.

### VETERINARY ASSOCIATION OF THE DISTRICT OF COLUMBIA

THE thirteenth meeting was held at Elks' Hall, March 27, 1897, at 8 P.M. All the leading veterinarians of the district were present. The minutes of the previous meeting were read and approved. One new member was elected.

The President appointed a Legislative Committee. consisting of Drs. Turner, Buckingham, and Pearson, to look after the interests of the bill to regulate the practice of veterinary medicine in the District of Columbia which failed to come up during the Fifty-fourth Congress.

The following officers were then elected for the ensuing year: President, Dr. H. Acheson; Vice-President, Dr. D. E. Buckingham; Secretary and Treasurer, Dr. J. P. Turner; Trustee, Dr. Pearson.

The Auditing Committee audited the Treasurer's account, after which several motions were approved relating to initiation fees and yearly dues.

A motion was made to call the next meeting on the last Saturday night in May, and thereafter on the last Saturday night of each alternate month. Approved.

Dr C. B. Robinson brought forward the subject of "Tuberculosis" for general discussion, paying especial attention to the use of the meat and milk of tuberculous animals, wishing this Association to take a definite position as to whether the meat or milk of any tuberculous animal, no matter how slightly diseased, should be permitted in the market. The subject brought forth a very interesting general discussion, which lasted until 11.30 P.M., when it was decided that each member should prepare a short article on the subject, quoting authorities, for the next meeting, when it is hoped a stand will be made on this very important subject.

Moved and seconded that the meeting adjourn. Approved.

J. P. TURNER,
Secretary.

